# Magnetofection Enhances Lentiviral-Mediated Transduction of Airway Epithelial Cells through Extracellular and Cellular Barriers

**DOI:** 10.3390/genes7110103

**Published:** 2016-11-23

**Authors:** Stefano Castellani, Clara Orlando, Annalucia Carbone, Sante Di Gioia, Massimo Conese

**Affiliations:** 1 Department of Medical and Surgical Sciences, University of Foggia, V. L. Pinto 1, 71122 Foggia, Italy; stefano.castellani@unifg.it (S.C.); annalucia.carbone@gmail.com (A.C.); sante.digioia@unifg.it (S.D.G.); 2 Brainlab AG, Kapellenstrasse 12, 85622 Feldkirchen, Germany; orlandoclara@libero.it

**Keywords:** airway epithelial cells, lentivirus, magnetofection, mucus, tight junctions

## Abstract

Gene transfer to airway epithelial cells is hampered by extracellular (mainly mucus) and cellular (tight junctions) barriers. Magnetofection has been used to increase retention time of lentiviral vectors (LV) on the cellular surface. In this study, magnetofection was investigated in airway epithelial cell models mimicking extracellular and cellular barriers. Bronchiolar epithelial cells (H441 line) were evaluated for LV-mediated transduction after polarization onto filters and dexamethasone (dex) treatment, which induced hemicyst formation, with or without magnetofection. Sputum from cystic fibrosis (CF) patients was overlaid onto cells, and LV-mediated transduction was evaluated in the absence or presence of magnetofection. Magnetofection of unpolarized H441 cells increased the transduction with 50 MOI (multiplicity of infection, i.e., transducing units/cell) up to the transduction obtained with 500 MOI in the absence of magnetofection. Magnetofection well-enhanced LV-mediated transduction in mucus-layered cells by 20.3-fold. LV-mediated transduction efficiency decreased in dex-induced hemicysts in a time-dependent fashion. In dome-forming cells, zonula occludens-1 (ZO-1) localization at the cell borders was increased by dex treatment. Under these experimental conditions, magnetofection significantly increased LV transduction by 5.3-fold. In conclusion, these results show that magnetofection can enhance LV-mediated gene transfer into airway epithelial cells in the presence of extracellular (sputum) and cellular (tight junctions) barriers, representing CF-like conditions.

## 1. Introduction

The airway epithelium represents the target of gene delivery vectors in inherited (e.g., cystic fibrosis(CF)) and acquired (e.g., asthma) lung diseases [1], however, extracellular and cellular barriers posit serious limits to the efficiency of this therapeutic approach. Among the extracellular barriers, the airway mucus is the most important one [2]. Although investigations made more than 10 years ago [3,4] demonstrated that pathological mucus impedes viral and nonviral gene transfer into airway epithelial cells, only recently has it been shown that the diffusion of both adenovirus and adeno-associated virus is strongly hindered in CF purulent mucus (i.e., sputum) [5]. Furthermore, poor gene transfer to the airway epithelium has been also attributed to limited cellular uptake across the apical membrane of the lung airway epithelium, inefficient trafficking to the nucleus, vector toxicity, and immunological barriers [6]. Tight junctions (TJs) can represent a formidable barrier to the entry of viruses into the airway epithelial cells. TJs restricts apical entry of viral gene transfer agents into the airway epithelium, since the expression of receptors for viral vectors is more abundant on the basolateral membrane than on the apical side of the respiratory epithelium, and they are hardly accessible because of the airway tight junctions [7,8,9]. Modification of the paracellular permeability and pseudotyping with heterologous envelopes are the strategy currently used to overcome the paucity or lack of viral receptors on the apical surface of the respiratory epithelium and to reach the basolateral membrane receptors [10,11]. However, the safety of these methodsis hampered by using toxic viral proteins or allowing, although transiently, the transepithelial passage of noxious products produced by bacteria and inflammatory cells.

Magnetofection (i.e., the nucleic acid delivery under the influence of a magnetic field acting on nucleic acid vectors that are associated with magnetic (nano)particles) has been used to improve the dose–response relationship in nucleic acid delivery and in the kinetics of the delivery process, as well as to localize nucleic acid delivery to an area which is under magnetic field influence [12]. This last issue is of extreme importance in the field of respiratory gene delivery, where the mucociliary clearance system removes particulates, such as nonviral and viral vectors, from the lung. Thus, the local confinement of nucleic acid delivery is an important requirement for gene delivery to the airway epithelium. Previously, we have shown that magnetofection can decrease the dose and the time of a lentiviral vector (LV) incubation with airway epithelial cells while maintaining a meaningful efficiency [13]. Furthermore, magnetofection resulted in at least 20-fold higher gene transfer levels than LV alone in a polarized model of airway epithelium.

In this study, we have approached the issue of enhancing LV-mediated gene delivery to the airway epithelium by magnetofection in the presence of extracellular and cellular barriers in two in vitro cellular models obtained with H441 bronchiolar epithelial cells (i.e., polarized cells overlaid with CF sputum supernatant (sol phase) and cultures forming hemicysts. In both models, we demonstrate that LV-mediated transduction can be aided in overcoming extracellular and cellular barriers by magnetofection. These results implicate that LV carrying the cystic fibrosis transmembrane conductance regulator (*CFTR*) gene could be targeted and concentrated at the level of CF bronchi/bronchioli by magnetofection, resulting in a higher pulmonary transduction without extrapulmonary expression.

## 2. Materials and Methods

### 2.1. Lentiviral Vector Production and Titration

The self-inactivating pRRLsin18.cPPT.CMV.eGFP.Wpre and pCCL.PPT.U1aENaCshRNA.hPGKΔNGFR.Wpre constructs were used to generate vesicular stomatitis virus (VSV)-pseudotyped LV stocks for green fluorescent protein (GFP)—LV–GFP and nerve growth factor receptor (NGFR) —LV–ΔNGFR, respectively—as previously described [14,15,16]. The viral titer was determined by HeLa cell (ATCC^®^ CCL-2™) infection and subsequent flow cytometry analysis, as previously described [14]. The yield of the concentrated virus was typically 10^9^ transducing units (TU)/mL and the specific activity ranged between 1.5 × 10^5^ and 4.17 × 10^5^ TU/ng of p24, a capsid protein of 24 kDa. The multiplicity of infection (MOI; TU/cell) used in three experiments is based on the titer (TU/mL) on HeLa cells, and the number of target cells.

### 2.2. Paramagnetic Nanoparticles

Magnetofection experiments were conducted by using two kinds of paramagnetic nanoparticles, ViroMag (VM) and ViroMag R/L (R/L) (OZ Biosciences, Marseille, France). The VM is a magnetic nanoparticle formulation dedicated to increase virus infection and transduction capacities, and it is suitable for all viruses. R/L is a magnetic nanoparticle formulation optimized for retroviruses and lentiviruses. The concentration of nanoparticles is estimated to be between 2.5 × 10^9^ and 5 × 10^9^/mL. ViroMag R/L has a mean size of about 350 nm with a zeta potential of +38 mV, whereas ViroMag has a mean size of about 160 nm (range from 160 to 220 nm) with a zeta potential of about +57 mV.

### 2.3. Transduction of H441 Cells with Different LV MOIs

In a first set of experiments, cells (1 × 10^5^) grown in a 24-well plate in Roswell Park Memorial Institute (RPMI)1640 medium containing 5% fetal bovine serum (FBS), 100 U/mL penicillin, and 100 μg/mL streptomycin (Sigma Aldrich, Milan, Italy) in RPMI, were incubated with the LV–GFP at different MOIs, and the GFP expression was evaluated 72 h after the infection. Since we seeded 1 × 10^5^ cells per well, multiplicity of infection equal to 10 (10 MOI) is equivalent to 1 × 10^6^ TU; 100 MOI refers to 1 × 10^7^ TU, and so on.

### 2.4. Magnetofection-Assisted Transduction of H441 Cells

Based on the LV MOI used in magnetofection experiments (i.e., 50) and the range of magnetoparticle concentration, the ratios between magnetic nanoparticles and LV virions were 2.5–5 for 2 μL; 7.5–15 for 6 μL; and 15–30 for 12 μL of ViroMag/ViroMag R/L.

Briefly, 7 × 10^4^ cells were plated on a 24-well plate in 500 μL of complete medium containing 10% FBS, l-glutamine and penicillin/streptomycin. On the following day, 50 μL of phosphate-buffered saline (PBS) containing LV (50 MOI) was added to 50 μL of PBS containing 2, 6, or 12 μL of ViroMag or ViroMag R/L and incubated for 15 min in order to form interactions between LV and magnetic particles. Then, 400 μL of complete medium was added and this mixture was dispensed onto the cell culture plate, which was either placed on top of the magnetic plate (OZ Biosciences, Marseille, France) or in the absence of magnetic field, and incubated for 20 min. LV particles were removed by washing two times with PBS 24 h later, then fresh complete medium was added. The GFP expression was evaluated after 48 h.

For time-course experiments, only 2 μL of VM was used. After different time points, cells were washed two times with PBS and, subsequently, complete medium was added. Cells were incubated at 37 °C 5%CO_2_ for 72 h, and then analyzed by flow cytometry (FACScan apparatus, Becton-Dickinson, San Jose, CA, USA) to evaluate efficiency, as previously described [13].

### 2.5. Polarization of H441 Airway Epithelial Cells

H441 cells were seeded onto 6.5 mm diameter Snapwell inserts (Corning, Acton, MA, USA) at 1.6 × 10^5^ per filter and grown overnight in RPMI 1640 medium supplemented with FBS (10%), l-glutamine (2 mM), sodium pyruvate (1 mM), insulin (5 μg/mL), transferrin (5 μg/mL), sodium selenite (10 nM), and antibiotics (penicillin/streptomycin) (Sigma Aldrich, Milan, Italy). The following day, the serum in the medium was replaced with 4% charcoal stripped serum (VWR International PBI, Milan, Italy), and the supplementations also included thyroxine (10 nM) and dexamethasone (200 nM), both from Sigma-Aldrich. From the third day of culture, cells were grown in presence of only medium in the basolateral compartment (i.e., at the air–liquid interface). Medium was changed daily for four more days. Under these conditions, cells grow as a polarized sheet of cells and develop a mean transepithelial resistance of 420 Ω × cm^2^, as measured by a volt-ohmmeter (Millicell-ERS; Millipore, Milan, Italy).

### 2.6. Transduction of Polarized H441 Cells in the Presence of CF Sputum Sol

Sputum was collected from four CF patients by spontaneous coughing and was immediately vortexed and then centrifuged for 5 min at 7000 × *g*. The supernatant (sol phase) was immediately removed and stored in aliquots at −80 °C. For transduction in the presence of CF sputum sol, polarized H441 cells were overlaid with 50 μL of the sol phase diluted 1:2 with RPMI 1640 complete medium (supplemented with penicillin/streptomycin) containing either LV alone (50 MOI) or LV preincubated with 2 μL VM. Cell monolayers were incubated in presence or in absence of magnetic field for 20 min and evaluated 72 h later. Unprocessed sputum determined detachment of cell sheets from the filter, and therefore was not used in these experiments.

### 2.7. Cell Culture and LV Transduction of H441 Cells Forming Domes

H441 cells were plated in a 96-well plate at a density of 30,000 cells/well in RPMI 1640 medium containing 5% FBS, 4.5 g/L glucose, 100 U/mL penicillin, and 100 μg/mL streptomycin. Dexamethasone was added daily for 5 days starting 2 days after seeding. The cells were infected with the LV–ΔNGFR1, 2, and 5 days after the first addition of dexamethasone at a MOI of 2000 and analyzed for transduction efficiency. Seventy-two hours post-infection, cells were detached by trypsin treatment, washed in PBS, and incubated with R-phycoerythrin (R-PE)-conjugated anti-NGFR antibody (BD Biosciences, Erembodegem, Belgium) for 30 min at 4 °C. Cells were centrifuged at 350× *g*, resuspended in PBS and analyzed by flow cytometry, evaluating the percentage of positive cells for FL2fluorescence (excitation 488 nm, emission 575 nm). The percentage of ΔNGFR^+^ cells was determined after setting the gating on 99% of an untransfected control population of cells and by subtracting the fluorescence of the untransfected control cells. Ten thousand cells were examined in each experiment. Analysis of ΔNGFR^+^ cells was performed by plotting the R-PE channel (FLH-2) against the FLH-3 channel, as previously done [16]. For the evaluation of GFP expression in domes, cells were infected with LV–GFP at 2000 MOI preincubated with 2 μL of VM in presence of magnetic field for 20 min. Then, cells were washed with PBS, incubated in complete medium and analyzed after 72 h using a Zeiss Axiovert 135 microscope equipped with GFP filter (excitation 395 nm, emission 509 nm).

### 2.8. Zonula Occludens-1 Localization

H441 cells grown on transwells for 6 days, in presence or in absence of 50 nM dexamethasone, were washed three times with PBS, fixed in 3% paraformaldehyde, and 2% sucrose and permeabilized with ice cold Triton Hepes buffer (20 mMHepes, 300 mM sucrose, 50 mM NaCl, 3 mM MgCl_2_, 0.5% Triton X-100, pH 7.4) for 5 min at room temperature. Cells were incubated with blocking solution (2% bovine serum albumin (BSA), 2% FBS), for 15 min at 37 °C, then with fluorescein isothiocyanate-conjugated mouse anti-zonula occludens-1 (ZO-1) antibody (Zymed Laboratories Inc., San Francisco, CA, USA) (dilution 1:125) for 30 min at 37 °C. Cells were rinsed three times with 0.2%BSA, and nuclei were counterstained with propidium iodide (0.1 μg/mL in PBS) for 5 min at room temperature. Filters were excised and placed side-up on a glass slide, and overlaid with a drop of Mowiol (Calbiochem, San Diego, CA, USA) followed by a coverslip. Cells were analyzed using a Zeiss Axioskop equipped with a laser scanning confocal unit model MRC-1024 (Bio-Rad, Hercules, CA, USA) containing a 15 mW krypton–argon laser. Specimens were viewed through a Planapo×63/1.4 oil immersion objective. Digital images were processed using Laser Sharp 2000 software (Bio-Rad, Segrate, Milan, Italy).

### 2.9. Statistical Analysis

Statistical significance of differences was evaluated by a two-tailed unpaired Student’s *t*-test or analysis of variance (ANOVA) using Fisher partial least-squares difference (PLSD) as posthoc test. Results were considered significant when *p* < 0.05.

## 3. Results

### 3.1. Magnetofection Enhances LV Transduction of Bronchiolar Epithelial Cells

**Figure 1:**
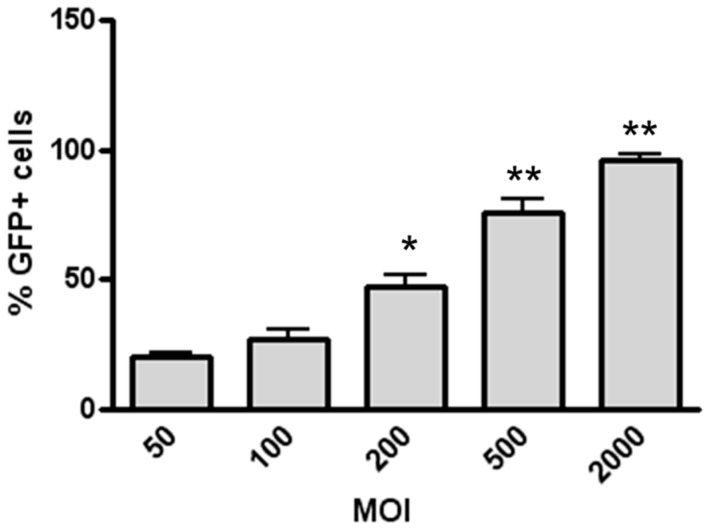
Efficiency of lentivirus–green fluorescent protein(LV–GFP) transduction in H441 cells. H441 cells grown in a 24-well plate were incubated with different multiplicities of infection (MOIs) of LV–GFP for 24 h, then medium was replaced and GFP expression was evaluated by flow cytometry 48 h later. Data are expressed as mean ± standard deviation (SD) of three experiments. * *p* < 0.05 for 200 MOI vs. 50 MOI; ** *p* < 0.01 for 500 MOI and 2000 MOI vs. 50 MOI.

We evaluated the transduction efficiency of a vesicular stomatitis virus-glycoprotein (VSV-G) pseudotyped lentiviral vector expressing GFP that can be detected by flow cytometry. First, we investigated whether the LV–GFP transduced H441 cells plated on plastic and forming a monostrate. With a low MOI (i.e., 50 MOI) as virion-to-cell ratio, the percentage of transduced cells summed up to about 20%, while 2000 MOI determined 96% of transduced cells (Figure 1).

**Figure 2:**
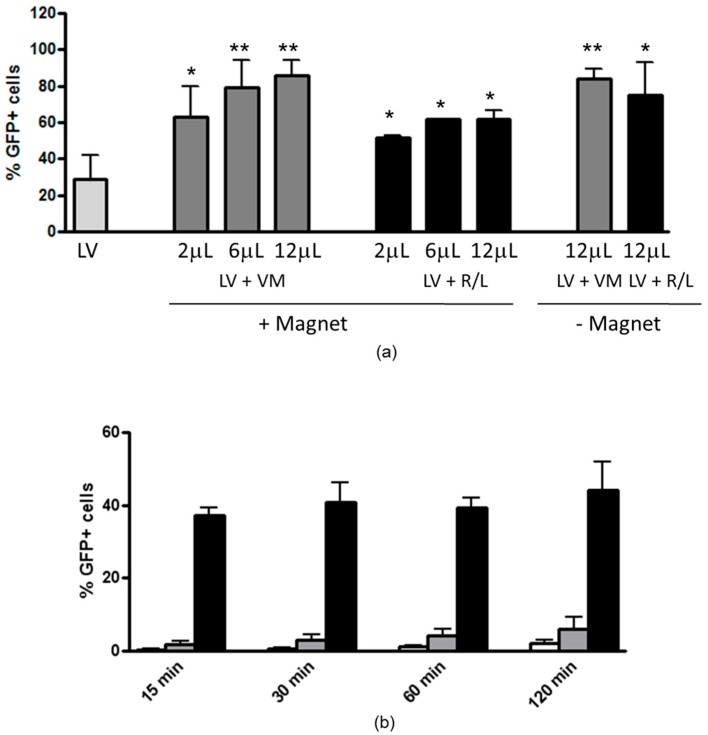
Effect of magnetofection on LV-mediated transduction efficiency of H441 airway epithelial cells, and time-course of LV-mediated transduction assisted by magnetofection. (**a**) H441 cells were incubated for 24 h with LV at a MOI of 50 alone or formulated as magnetic vectors with three different doses of ViroMag (VM, gray bars) or ViroMag R/L (R/L, black bars) magnetic particles, in the absence or in the presence of a magnetic field. Cells were exposed to magnetic field for 20 min. LV particles were removed 24 h later and the GFP expression was evaluated after 48 h by fluorescence-activated cell sorting(FACS). Data are expressed as the mean ± SD of three experiments. * *p* < 0.05; ** *p* < 0.01 (magnetofectins vs. LV alone); (**b**) H441 cells were incubated with LV alone at a MOI of 50 (white bars), or formulated as magnetic vector with 2 µL of magnetic particles, in the absence of magnetic field (gray bars) or in the presence of magnetic field (black bars). Cells were exposed to magnetic field for 15 min and then, after the removal of the magnet, LV particles were washed off at 0, 30, 60, and 120 min and GFP expression was evaluated 72 h later. Data are expressed as the mean ± SD of three experiments. All conditions with magnetofectins were significantly different compared to LV alone, with a *p* value<0.0001.

Next, we studied if paramagnetic particles can enhance LV-mediated gene expression subsequent to transduction. Virions were incubated with either VM or R/L particles in PBS to form magnetofectins and then added to the cells. The transduction efficiency increased in a dose-dependent fashion with up to a 2.9-fold increase with VM-containing magnetofectins (Figure 2a). Interestingly, 12 μL of VM magnetoparticles were able to enhance lentiviral transduction with 50 MOI approaching the levels obtained with 500 MOI. With R/L magnetoparticles, the enhancement effect was up to 2.1-fold. As noticed before with other respiratory epithelial cell lines [13], even in the absence of magnetic forces, the highest dose of magnetoparticles used (12 μL) was able to give an enhancement effect of 2.8- and 2.5-fold with VM and R/L, respectively. The following experiments were carried out with lentiviral magnetoparticles obtained with 2 μL of VM, since 12 μL had resulted in enhanced cytotoxicity in our previous study [13].

### 3.2. Magnetofection Can Reduce the Time of Contact between LV and Cells to Obtain Efficient Transduction

To determine whether magnetofection could shorten the time of contact between virions and cells without influencing the transduction efficiency, magnetofectins were incubated with cells for different time points up to 2 h, in presence or in absence of a magnetic field. Cells were then extensively washed and incubated for 72 h to allow GFP expression. Magnetofection significantly increased the percentage of GFP+ cells as quickly as 15 min after start of incubation compared not only to LV alone, but also to magnetofectins in the absence of magnetic field (Figure 2b). With only 15 min of incubation, the LV-mediated transduction was increased by 81-fold by magnetofection with VM in comparison with LV alone, and this effect progressively decreased as a result of the increment in cells transduced by the LV alone (the increase was 21.2-fold after 120 min of incubation).

### 3.3. Magnetofection Enhances LV-Mediated Transduction through CF Sputum Sol

**Figure 3:**
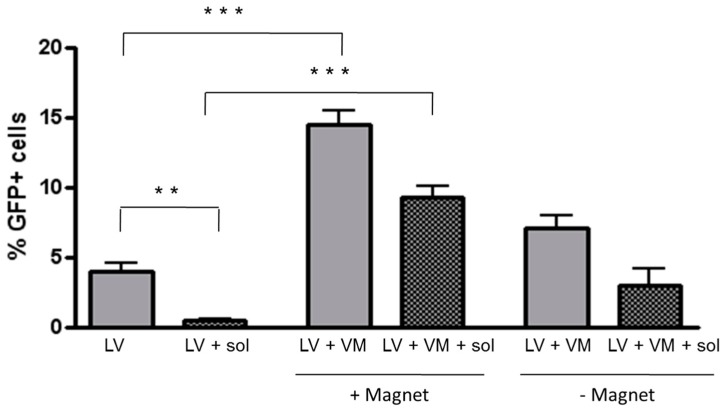
Effect of magnetofection on efficiency of LV transduction in presence of CF sol. Polarized H441 cells grown for 6 days on filters were layered with CF sputum sol on their top and infected with LV alone (50 MOI) or combined with VM in presence or in absence of magnetic field, and analysed 72 h later. The percentage of transduced cells was significantly lower than cells transduced in the absence of sputum. The transduction of sputum-overlaid cells with LV–VM in the presence of magnetic field was found to be higher in comparison with LV alone and LV–VM in the absence of magnetic field, respectively. Data are expressed as the mean ± SD of three experiments. *** *p* < 0.0001 for LV+VM in presence of magnetic field vs. LV alone both in presence or in absence of sol; ** *p* < 0.001 for LV+sol vs. LV alone.

These results showed that magnetofectins can enhance LV-mediated transduction of H441 cells and prompted us to analyze whether this effect could be obtained also in the presence of mucusCells, when overalaid with unprocessed sputum for 24 h, detached from the substratum (not shown). For this reason, we used the sputum sol phase separated from the gel phase by centrifugation, which has been demonstrated to be nontoxic to cells. H441 were grown on Transwell filters in order to allow their polarization. When cells grown for 6 days on filters that were layered with CF sputum sol on top, the percentage of transduced cells was significantly lower than cells transduced in the absence of sputum, with a reduction of almost 8.7-fold (Figure 3). The transduction of sputum-overlaid cells with LV–VM in the presence of magnetic field was found to be 20.3- and 3.1-fold higher in comparison with LV alone and LV–VM in the absence of magnetic field, respectively.

### 3.4. Dexamethasone Treatment Increases Tight Junction Organization and Induces Refractoriness to LV-Mediated Transduction

**Figure 4:**
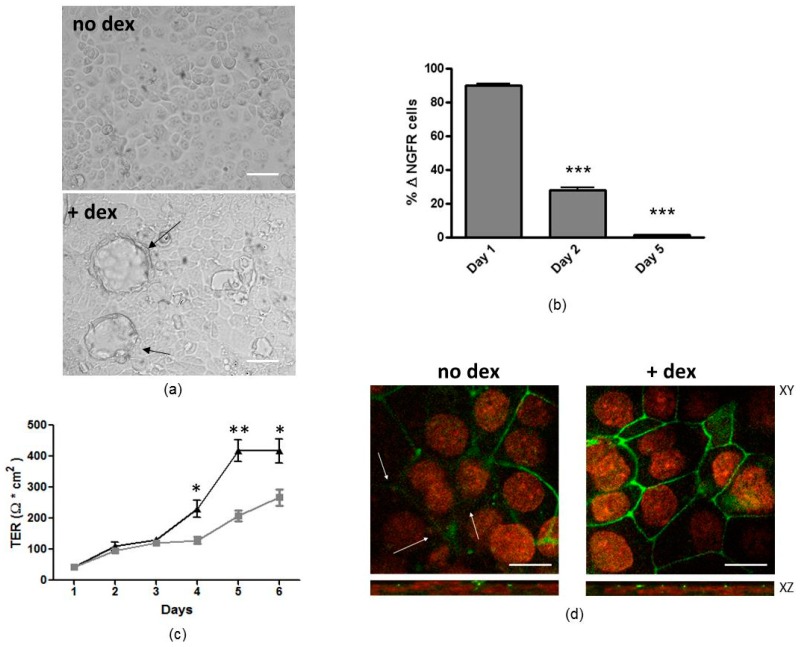
Effect of dexamethasone treatment on dome formation, transepithelial resistance (TER), zonula occludens-1 (ZO-1) organization, and efficiency of LV transduction. (**a**) Light microscopy images show that dexamethasone treatment of H441 cells (+dex) resulted in the formation of fluid-filled hemicysts (“domes”), which appear from the third day of treatment, indicated by black arrows. Cells without treatment are shown as negative control (no dex). Scale bar = 40 μm; (**b**) H441 cells were infected with LV–∆NGFR (a lentivirus encoding for nerve growth factor receptor) at 2000 MOI at day 1, 2, or 5 of treatment with dexamethasone, and ∆NGFR expression was evaluated 72 h later by flow cytometry. Data are expressed as the mean ± SD of three experiments. *** *p* < 0.0001 for efficiency of transduction at day 2 and 5 of dexamethasone treatment vs. efficiency obtained at day 1 of dexamethasone treatment; (**c**) H441 cells plated on transwells were treated daily with dexamethasone (black triangles) or not treated (gray squares). Dexamethasone increased TER from the third day of treatment. Data are expressed as the mean ± SD of four experiments. * *p* < 0.05; ** *p* < 0.001 for dex-treated cells vs. untreated cells; (**d**) ZO-1 localization (green signal) was evaluated by confocal microscopy in cells treated daily or not treated with dexamethasone. Nuclei were counterstained with propidium iodide in red. White arrows indicated points of discontinuous ZO-1 junctional staining in the absence of dexamethasone (no dex), whereas its staining was stronger and present at all intercellular borders after 5 days for dexamethasone-treated cells (+dex); xy, horizontal section; xz, vertical section. Scale bar = 10 μm.

H441 cells form fluid-filled hemicysts, called “domes”, when grown in presence of 50 nM dexamethasone, which appear from the third day of treatment (Figure 4a). The transduction efficiency of the LV was assayed in H441 cultures induced with dexamethasone, which were forming domes. In preliminary experiments, we used 50 MOI with untreated cells, and∆NFGR expression was not detectable (not shown). Thus, transduction was performed with a higher virion-to-cell ratio (i.e., 2000 MOI). By increasing time of treatment with dexamethasone, H441 became progressively more resistant to infection, even at higher MOI of 2000 (1.43% ± 0.17% of positive cells at day 5 of treatment) by using LV–∆NGFR (Figure 4b).

To confirm whether a higher organization of tight junction was a feature of dexamethasone-treated H441, transepithelial resistance (TER) and ZO-1 localization were studied in polarized H441 cultures. Figure 4c shows the increase in TER as a function of incubation time with dexamethasone up to 6 days, while absence of dexamethasone treatment resulted in a significantly less pronounced increase of TER. ZO-1 showed a discontinuous junctional staining and was not present at every intercellular junction in H441 cells cultured in the absence of dexamethasone (Figure 4d), whereas its staining was stronger and present at all intercellular borders in dexamethasone-treated cells (Figure 4d).

### 3.5. Magnetofection Increases LV Transduction of Epithelial Domes

**Figure 5:**
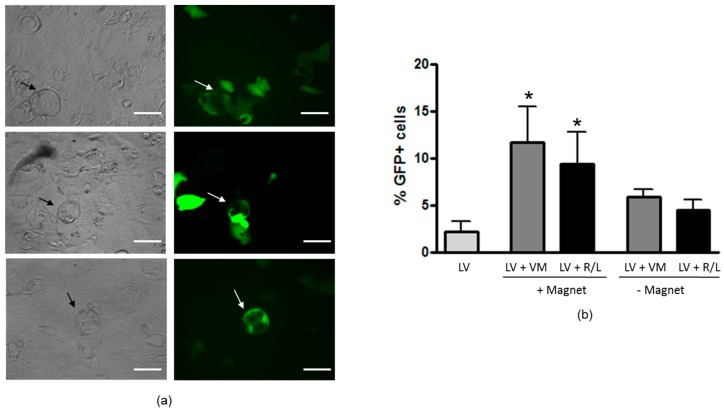
Effect of magnetofection on dome transduction by LV–GFP. (**a**) GFP expression in H441 cells forming domes after LV–GFP magnetofection-assisted infection. Left panels show bright fields (black arrows indicating domes); right panels show epifluorescence of the same fields (white arrows indicating transduced domes). Original magnification: 32×. Scale bar = 40 μm; (**b**) H441 cells were infected with LV at 2000 MOI combined with 2 μLVM or R/L in presence or in absence of magnetic field. GFP expression was evaluated 72 h post-infection. Data are expressed as the mean ± SD of three experiments * *p* < 0.05 for LV+VM or LV+R/L in presence of magnetic field vs. LV alone.

In order to visually localize transduced cells, magnetofection was carried out on dome-forming cells with an LV–GFP. The GFP expression was detected mainly in epithelial domes and, to a lesser extent, in surrounding cells (Figure 5a). The percentage of GFP+cells was lower as compared to nontreated cells and was found at very low levels (2.2% ± 1.1%) (Figure 5b). Magnetofectins used in the absence of the magnetic field did not significantly enhance transduction efficiency as compared to LV alone, whereas in the presence of the magnetic field there was a significant increase with both VM and R/L magnetoparticles (11.7 ± 3.9 and 9.7 ± 3.3, respectively).

## 4. Discussion

Magnetofection has some advantages when compared with other physical methods of gene delivery (electroporation, sonoporation, etc.); that is, it combines simplicity, cost-effectiveness, localization of delivery, enhanced efficiency, and reduction of incubation time and of vector doses [12,17]. In the field of gene therapy, magnetofection has been a widely used research tool, allowing transfection/transduction of primary cells and organs in vivo. Magnetofection has been used in the field of respiratory epithelial cells to increase nonviral-mediated gene transfer [18,19,20,21] and to study the mechanism of internalization [22]. Although many studies have demonstrated that by using viral magnetofection systems, the expression of a transgene is often significantly higher in comparison to only inoculating with virus alone in many cell types [23,24,25,26,27], our previous work was the first to show that LV-mediated transduction of bronchial epithelial cells is enhanced by magnetofection [13]. Moreover, only a few studies have demonstrated the usefulness of magnetofection in targeting applications that require penetration of a cellular or a tessutal barrier [28]. For example, Zhang et al. have employed superparamagnetic iron oxide nanoparticles to condense silencing RNAs and plasmids to deliver nucleic acids in cells embedded in a 3D collagen matrix and have shown that magnetic vectors penetrate the matrix under magnetic field influence and transfect cells [29]. MacDonald and colleagues have shown that a time-varying magnetic field greatly improves the transport of magnetic nanoparticles in a viscous gel, likely by reducing the effective viscous drag acting on carriers [30]. Notably, to the best of our knowledge, no study has been performed in order to understand the enhancing effect of magnetofection on the lentiviral transduction of respiratory epithelial cells in the presence of extracellular and cellular barriers.

In this paper, we have found that magnetofection enhances LV-mediated transduction of bronchiolar epithelial cells in a dose- and time-dependent manner (Figure 2). Similar to what we found with another airway epithelial cell line (i.e., 16HBE bronchial cells [13]), the transduction in the absence of a magnetic field led to some enhancement, which was not significant. However, high transduction levels can be associated with increased cytotoxicity. For this reason, the other set of experiments were carried out in more complex models with fewer magnetoparticles. Nevertheless, also under these experimental conditions, we obtained an enhancement of tranduction efficiency over the LV alone.

The respiratory mucus represents the most important barrier to gene transfer into airway epithelial cells. This correlates well with previous findings demonstrating low diffusivity of viral vectors into the CF mucus [5]. The CF sputum sol is a good model for intact CF sputum since the content in phospholipids [31] and mucins (such as MUC5AC and MUC5B) [32] is very similar. Moreover, the CF sputum sol phase has been shown to inhibit adenovirus-mediated gene transfer in cultured epithelial cells [33], although this result was due to the presence of preexisting antibodies rather than to steric hindrance by CF sputum components. Nevertheless, our data are in agreement with these previous studies:the LV-mediated transduction was decreased in the presence of the CF sputum sol. Previously, we have shown that in the absence of polarization and incubating 16HBE bronchial cells for periods longer than 4 h, magnetofectins alone (i.e., in the absence of a magnetic field) gave an enhancing effect on LV-mediated transduction, an effect that is likely the result of the settling of magnetofectins upon the cells by gravity. Magnetofectins were unable to give this effect in the absence of a magnetic field when the experiments were performed for short times with either nonpolarized or polarized cells. The concern arose that this could limit the application of the magnetofection technique in vivo as a result of the thickness of tissues that could oppose the magnetic forces at the level of the respiratory epithelium, a setting that could necessitate more than 20 min of incubation time. However, it must be said that in vivo experiments in mice have demonstrated the feasibility of magnetofection to the lung, studied either withplasmid DNA (pDNA) deposition [34] or luciferase expression [35], when an external magnetic field was applied externally on the thorax (up to 72 h [35]). Interestingly, in the present study, magnetofection increased the transduction levels in the presence of the sol phase obtained from CF sputum, a phenomenon which was not reproducible in the absence of a magnetic field (Figure 3). The mechanism underlying this effect is not known at the moment. This model does not contemplate the mucociliary clearance and the horizontal transfer [36], while only vertical transfer is under study. However, data presented in Figure 2, obtained by washing off the LV, could mimic what happens in vivo with the mucociliary clearance. Overall, our conclusion is that magnetofection increases magnetic virion transport through the sol layer by impeding various adhesive interactions which may occur between viral vectors and sputum constituents [5], and by allowing a firm adhesion to the airway epithelium. One limitation of the present work is the impossibility to use unprocessed CF sputum since it is toxic to the cells, as such (i.e., induced cell detachment from the substrate). Only one previous study has determined the inhibition of adenovirus-mediated gene transfer after incubation of respiratory epithelial cells with unprocessed CF sputum [3], although the authors from this study did not discuss the cytotoxicity of the CF sputum. To reconcile these contrasting results, which should be deepened in further work, other artificial systems that integrate mucins, DNA, and actin from extravasated neutrophils should be used as previously published [37]. Moreover, further studies should be conducted with fluorescently labeled virions to understand the fate of magnetic virions within the native mucus or sputum sol phase.

Dexamethasone-induced dome formation is caused by progressive development of tight junctions in H441 cells, which allows vectorial epithelial Na^+^ channel (ENaC)-dependent sodium transport, at the apical membrane [38,39]. This drives osmotic fluid transport to the basolateral compartment [38,39]. These data are in good agreement with those published by Sekiyama et al., which demonstrated that dexamethasone increased TER and ZO-1 and occludin organization at the level of cell–cell contact in airway epithelial cells [40]. ZO-1 is one of the major TJ proteins and is involved in TJ structure and function as well as in cytoskeletal organization of the epithelia [41]. What is interesting from our results is that LV-mediated transduction was enhanced by magnetofection at the level of epithelial domes and, to lesser extent, in cells surrounding the domes (Figure 5). These data indicate that cells not forming domes are more resistant to magnetofection-assisted transduction. The increase in ZO-1 localization at the intercellular borders in dex-treated cells is in agreement with this finding (Figure 4), indicating that TJs can impose an important barrier to magnetofection-aided LV-mediated gene transfer. Why the domes seem to be more permissive than isolated cells is not known at the moment. Shlyonsky et al. showed that the amiloride-sensitive, selective membrane Na^+^ conductance could only be detected in cells that formed these domes, whereas cells located away from these structures expressed the La3^+^-sensitive, nonselective cation conductance [38]. Thus, it could be that differences in permeability can also exist between dome-forming cells and cells out of domes. In this case, cells forming domes might permit more allowance to receptors and/or molecules aiding viral magnetofectins attachment and entry expressed on the basolateral membrane. Further studies should be carried out to investigate more precisely TJ organization and function (i.e., permeability) at the level of domes, justifying increased transduction levels with magnetofection.

## 5. Conclusions

The main goal of this work was to understand whether extracellular and cellular barriers, represented by sputum and tight junctions, respectively, could be overcome by magnetofection-assisted LV transduction of airway epithelial cells. In regard to chronic respiratory diseases, whose airway lumen is filled with sticky mucus, these results may offer a novel avenue to transduce the airway epithelium, even in vivo. However, the usefulness of magnetofection in animal models whose airways are replenished with pathological mucus has not been established yet, and should be the focus of future work.
